# Molecular docking analysis of long-chain alkanes with the β-lactamase BEL-1 from *P. aeruginosa*

**DOI:** 10.6026/97320630018460

**Published:** 2022-05-31

**Authors:** VR Gayathri, B Prakash, JV Yashika, Subash Vetri Selvi, D Jegadeesh Kumar

**Affiliations:** 1 Department of Biotechnology, Vivekanandha College of Arts & Science for Women (Autonomous) Trichengode, Tamilnadu, India; 2 Department of Biotechnology, Vels Institute of Science Technology & Advanced Studies, Chennai, Tamil Nadu, India; 3 Electroanalysis and Bioelectrochemistry Lab, Department of Chemical Engineering and Biotechnology, National Taipei University of Technology, Taipei 106, Taiwan, ROC; 4 Chromopark Research Centre, Namakkal, Tamilnadu, India

**Keywords:** β-lactamase BEL-1, P. aeruginosa, nonadecane, long-chain alkanes

## Abstract

Pseudomonas aeruginosa is a gram-negative opportunistic bacterium that is a concern worldwide due to its innate antibiotic resistance properties. This warrants the development of non-antibiotic compounds that can potentially address the growing concern. Therefore, it is of interest to document the molecular docking analysis data of three long-chain alkanes, namely, eicosane, triacontane, and nonadecane with β-lactamase BEL-1. Data shows that nonadecane have good binding features and drug-likeness when compared to triacontane and nonadecane. It is well known that nonadecane is a compound that is abundantly available from natural resources for further consideration.

## Background:

Pseudomonas aeruginosa is an opportunistic gram-negative bacterium that can be seen along with nosocomial infections [1]. The genome sequence of P. aeruginosa is relatively large (5.5-7 Mbp) [2]. They are resistant to external stress, colonise easily, and rapidly develop a matrix of biofilm. P. aeruginosa consists of resistance-nodulation division efflux pumps which protect against antibiotics [3, 4]. The biofilm formed also provides resistance to antibiotics. P. aeruginosa strains have multiple drug-resistant [5]. A clinical strain of P. aeruginosa consisting of clavulanic acid-inhibited ambler class A extended-spectrum β-lactamase (BEL-1) was reported [6, 7]. BEL-1 is chromosomally encoded in class 1 integrons and has an association with cephalosporinase and penicillinase and it shows resistance against β-lactam antibiotics, including carbapenems [6,8]. The development of BEL-1 strain containing P. aeruginosa is a concern amidst the growing multi drug resistance bacterial species. Besides, the BEL-1 strain containing P. aeruginosa have low MIC and is sparsely detectable with conventional methods [9]. The World Health Organisation listed carbapenem resistant P. aeruginosa as a bacterial species that requires the development of a novel class of antibiotics for treatment [10]. New class of antibiotics will become vulnerable to the antibiotic resistance properties of the bacterium [11]. Non-antibiotic compounds with features such as inhibition of quorum sensing and bacterial lectins, phage therapy, vaccines, nanoparticles, etc are known [12,13]. There are several research groups working on developing novel therapies from naturally available resources such as fungi, plants, etc [14- 17]. Therefore, it is of interest to document the molecular docking analysis data of plant derived three long-chain alkanes, namely, eicosane, triacontane, and nonadecane with β-lactamase BEL-1.

## Materials and Methods:

### Protein preparation:

Molecular structure reported at the Protein Data Bank (PDB) was retrieved for extended-spectrum β-lactamase BEL-1 (PDB ID: 5EUA). The structure was energy minimised and converted to PDBQT format.

### Ligand preparation:

The compounds eicosane, triacontane, and nonadecane were selected as ligands for docking and their 3D structures were taken from PubChem. It was then minimized by applying Gasteiger and Kollman's charge. The root of the structure was detected, torsion was applied, and the structures were saved in PDBQT and Mol2 format.

### Molecular Docking and binding profile:

Molecular docking analyses were carried out using PyRx - Python Prescription 0.8. The compounds were targeted to the protein with a grid-box docking model to determine the binding energies. Furthermore, the interaction between the amino acids and compounds was identified using Cresset Flare 4.0.1. Besides, clogP, PSA, TSA, TPSA, drug-likeness, etc., were determined using Osiris Data warrior 5.5.0.

## Results and Discussion:

Phytoactive compounds such as eicosane, triacontane, and nonadecane that are identified in M. acumanata acetone extract were used in the docking analysis to understand the binding activity, binding energy and inhibitory activity against β-lactamase BEL-1 of P. aeruginosa. Figures 1 shows the docking of proteins and ligands created using PyRx - Python 0.8. The active site binding regions of the ligands were identified using Cresset Flare 4.0.1. The study revealed that eicosane has a binding energy of -5 kcal/mol and triacontane has -5.1 kcal/mol and nonadecane has -5.9 kcal/mol. The active binding site of eicosane is ILE 166, GLN 199 and for triacontane it is GLU 84, and GLN 117, and for nonadecane it is GLN 208, GLU 114, GLN 117, and GLN 121. Table 1(see PDF) gives the key parameters of analysis such as clogP, TSA, TPSA, drug likeness, etc.

Data shows that the compounds have considerable binding features with the antibiotic resistance β-lactamase BEL-1 from P. aeruginosa, with a drug-likeness value of -23.454 for nonadecane and -20.398 for both eicosane and triacontane. Moreover, the compounds did not have any mutagenic or tumorigenic activity. The compounds also obey Lipinski's rule of five. Eicosane, triacontane, and nonadecane are long-chain alkanes found in essential oils. The compounds exhibit both anticancer and antimicrobial activity. GC/MS study on Scenedesmusobliquus diethyl ether extract revealed show 76.10% of nonadecane with low activity against P. aeruginosa NRRL B-272 strain [18]. The extract from Ribesnigrum leaves consisting of a mixture of alkanes, especially nonadecane, shows the presence of activity against P. aeruginosa strain PA14 biofilm with no significant difference when compared to a positive control [19]. Allium chinense leaf extract showed a significant antimicrobial effect against P. aeruginosa (MTCC741) which is highly resistant to antibiotics [20]. Allium chinense have the presence of mainly three compounds, nonadecane, tetratetracontane, and 2-methyl-octadecyl trifluoroacetate. Moreover, essential oils from Rosa amascena Herrm show the presence of nonadecane in abundance. Essential oil consisting of nonadecane significantly inhibited the growth of P. aeruginosa (ATCC 27,833) [21]. Therefore, it is of interest to document the molecular docking analysis data of three long-chain alkanes, namely, eicosane, triacontane, and nonadecane with β-lactamase BEL-1. Data shows that nonadecane have good binding features and drug-likeness when compared to triacontane and nonadecane.

## Conclusion:

M. acumanata flower is commonly consumed in India. It has several medicinal properties. Studies show potential antibacterial and anticancer activities from this flow. Hence, we document the molecular docking analysis data of long-chain alkaneseicosane, triacontane, and nonadecane, with the BEL-1 of P. aeruginosa for further consideration with nonadecane shows optimal binding. Nonadecane is abundantly available and can be developed as potential non-antibiotic therapeutics to treat antibacterial infections, especially P. aeruginosa.
